# Barriers and incentives to the production of bioethanol from cereal straw: A farm business perspective

**DOI:** 10.1016/j.enpol.2013.03.003

**Published:** 2013-08

**Authors:** N.J. Glithero, S.J. Ramsden, P. Wilson

**Affiliations:** Division of Agricultural and Environmental Sciences, School of Biosciences, University of Nottingham, Sutton Bonington Campus LE12 5RD, United Kingdom

**Keywords:** Cereal straw, Bioenergy, Second generation biofuels

## Abstract

The EU renewable energy directive stipulates a requirement for 10% of transport fuels to be derived from renewable sources by 2020. Second generation biofuels offer potential to contribute towards this target with cereal straw representing a potentially large feedstock source. From an on-farm survey of 240 arable farmers, timeliness of crop establishment and benefits of nutrient retention from straw incorporation were cited as reasons for straw incorporation. However, two-thirds (one-third) of farmers would supply wheat (barley) straw for bioenergy. The most popular contract length and continuous length of straw supply was either 1 or 3 years. Contracts stipulating a fixed area of straw supply for a fixed price were the most frequently cited preferences, with £50 t^−1^ the most frequently cited minimum contract price that farmers would find acceptable. Arable farmers in England would be willing to sell 2.52 Mt of cereal straw for bioenergy purposes nationally and 1.65 Mt in the main cereal growing areas of Eastern England. Cereal straw would be diverted from current markets or on-farm uses and from straw currently incorporated into soil. Policy interventions may be required to incentivise farmers to engage in this market, but food and fuel policies must increasingly be integrated to meet societal goals.

## Highlights

• English arable farmer survey to determine potential supply for straw based biofuel.
• Two-thirds of farmers would supply wheat straw for bioenergy.
• Farmers willing to sell 1.65 Mt of cereal straw from the main cereal producing regions.
• Farmer preference for a fixed area of straw supply for a contracted fixed price.
• £50 t^−1^ the most frequently cited minimum contract price farmers find acceptable.

## 1 Introduction

As part of the drive to increase renewable energy use within Europe, the EU has set a revised target for 10% of total transport fuels to be derived from renewable sources by 2020 [EU, Directive 2009/28/EU]. In the UK, the main renewable transport fuels are biodiesel and bioethanol; much of the bioethanol is imported and derived from ‘first generation’ technologies (Bomb et al., 2007). The UK has implemented a range of policies to support renewable energy (see, for example, Mitchell and Connor, 2004) and more recently funding has been made available for research into ‘second generation’ fuel technologies (Anon, 2012a) i.e. those not based on crop products that have an alternative use as food for direct (or indirect) human consumption. As part of this research focus on second generation fuels, the Biotechnology & Biological Sciences Research Council (BBSRC) established ‘BSBEC’, the BBSRC Sustainable BioEnergy Centre (Anon, 2012b). Work within the Centre includes research into the lignocellulosic conversion of cereal straw into bioethanol. Bioethanol from this agricultural residue feedstock, as a ‘co-product second generation biofuel’ (CPSGB; Glithero et al., 2012), mitigates some of the concerns that have been raised in relation to land use change, as the use of a co-product does not compete directly with food production (Londo et al., 2010; Naik et al., 2010; Nigam and Singh, 2011; Williams, 2008). However, while some authors have argued that straw should be used as a replacement for fossil fuels in bioenergy production more generally (e.g. Gabrielle and Gagnaire, 2008), others have raised ‘sustainability’ concerns (Thornley et al., 2009); these include the potential depletion of soil organic matter if straw is not incorporated into the soil (Cherubinia and Ulgiatib, 2010; Lal, 2008). Set against this, it is worth noting that, before the UK straw and stubble burning ban of 1993, up to 41% of wheat straw in England and Wales was burnt in the field (Silgram and Chambers, 2002).

It has been estimated that on arable farm types in England 3.82 Mt of cereal straw (from wheat and barley) is currently used on-farm or sold, with a further 1.45 Mt chopped and incorporated into the soil (Glithero et al., in press). Another estimate puts the ‘straw surplus’ (from cereal crops chopped and incorporated), in Great Britain, from all farm types, at 5.7 Mt in 2007 (Copeland and Turley, 2008). In the UK, the Ely Combined Heat and Power plant (270 GWh plant) uses 200 kt of straw per annum and describes itself as the largest straw burning plant in the world (Anon, 2012c); in other countries considerable interest in using straw as an energy source is developing (Skött, 2011). The UK bioenergy strategy (Anon, 2012a) noted that in 2009 approximately 3% of UK cereals were converted into biofuels, using mainly first generation technology, generating 0.6 TWh of energy. The strategy also suggests that the ‘tradable surplus’ of UK cereals could be used for bioenergy production and that domestic supply of bioenergy feedstocks could produce over 75 TWh of energy from agricultural residues such as straw and dedicated biomass crops such as short rotation coppice (SRC) and miscanthus, as well as other biomass sources (e.g. managed woodland). Despite these positive estimates of feedstock supply for second generation technologies, a number of barriers to their use for bioenergy remain. We briefly consider the latter, below.

The potential reluctance of farmers to displace conventional cropping with dedicated energy crops has been noted by Convery et al., 2012. Although CPSGBs such as cereal straw do not lead to crop substitution, the co-products do have many alternative end uses. These include: animal bedding (Wolf et al., 2010), animal feed (Copeland and Turley, 2008), on-farm production of other crops (Döring et al., 2005), industry (such as burning for energy; Anon, 2004), crafts, such as thatching (Yates, 2006), building materials (Swanston and Newton, 2005), export (Tasker, 2011), and incorporation into soils providing potential soil organic matter enhancements and some nutrient supply to the following crop (Anon, 2010; Nicholson et al., 1997; Powlson et al., 2011). Of these, the majority of straw is used in livestock production or is incorporated. Additional barriers to the use of straw for bioethanol, beyond its current uses, are the costs and difficulty of storage and transportation over long distances (Swanston and Newton, 2005). Despite the wide range of potential end uses, a substantial proportion of straw in the UK, particularly in areas that are distant from livestock production, is currently chopped and incorporated. A major benefit of straw incorporation is improved timeliness of farm operations: incorporation allows more prompt establishment of the following crop (Darby and Yeoman, 1994). Machinery, storage and labour costs are also lower. As noted, straw incorporation has also been linked to improved soil organic matter levels in soils; indeed, the UK Code of Good Agricultural Practice (Anon, 2009a) states that: ‘*Incorporating crop residues that do not contain much nitrogen*, *such as cereal straw*, *into the soil in autumn will help to reduce the amount of nitrate leached and to maintain or increase soil organic matter*’.

Mitchell and Connor (2004) note bioenergy policy incentives at both industrial and feedstock supply levels and suggest that there is substantial potential for energy crops and agricultural waste products to be used in energy production in the UK. However, no UK or EU-wide policies related to straw removal for bioethanol or bioenergy purposes currently exist, which is in direct contrast with dedicated energy crops such as SRC and miscanthus, where, for example, the ‘Energy Crops Scheme’ (Anon, 2009b) in England provides crop establishment funding for SRC and miscanthus, albeit that several authors have also identified barriers towards dedicated energy crop uptake (Piterou et al., 2008; Sherrington et al., 2008; Sherrington and Moran, 2010; Alexander et al., 2011; McCormick and Kåberger, 2007).

Farmer decision making in relation to crop or enterprise choice and business activities is influenced by a wide range of factors (Edwards-Jones, 2006). Whilst historically farmers have been partially protected against the vagaries of the open market, through national and European support mechanisms (Gorton et al., 2008), they now operate in a much freer market environment, with attendant risks and opportunities, responding to market signals (Lobley and Butler, 2010). Cereal farmers can manage this environment, to an extent, by marketing their grain using a range of methods: forward contracts (an agreed price, quality and date for future sale), sale and purchase of futures contracts to hedge against falling prices, and ‘options’ which allow, at a premium, a farmer to both hedge against grain price falls and take advantage of upside market opportunities. Alternatively they may market all or some of their grain on the open ‘spot’ market.

Cereal straw is typically marketed on the spot market, via auctions or private sales and additionally as baled produce, ‘sold-in-swath’ (sold to a third party straw harvesting and transportation contractor or other farmer undertaking these functions)^1^ or sold as a standing crop. However, for bioenergy purposes, where large scale investment is needed on behalf of fuel producers, securing sufficient supply in a defined geographical region is likely to require contractual agreements with farmers, perhaps similar to those used for grain, to secure feedstock supply; currently there is no information on the characterisation of such contracts that farmers would find acceptable, nor the volume of straw that will potentially be supplied.

It is clear that, although cereal straw is a ‘co-product’, it has a range of potential benefits in its current uses, both as an end product and when incorporated into agricultural soils. The focus of the remainder of this paper is therefore an assessment of cereal straw supply for bioenergy production examining the barriers that exist at the farm-level with respect to supply of straw for bioenergy, as well as the incentives required to establish a sustainable feedstock supply base. The aim of the paper is to (a) describe the survey methodology used, (b) estimate the amount of straw that farmers would sell for bioenergy purposes, (c) indicate the number of years that farmers would supply straw and, in addition, the contractual aspects of supplying straw for bioenergy production that farmers would find acceptable, (d) illustrate potential barriers to feedstock supply for bioenergy in relation to straw, (e) examine regional logistic aspects of feedstock supply and (f) place these survey findings in the context of CPSGBs. The survey methodology is outlined in Section 2 along with the data analysis methods employed within the paper. The survey results in relation to contracts, amounts of straw and potential barriers to supply are presented in Section 3. Discussion of the survey findings in relation to bioenergy and in particular CPSGBs is given in Section 4 with concluding remarks in Section 5.

## 2 Method

### 2.1 Survey methodology and scope

A survey was undertaken to gain information relating to contract implications of bioethanol feedstock production, dedicated bioenergy crops, straw use, straw volumes baled, crop cultivations, cereal variety choice and straw incorporation. The survey questionnaire was designed with a variety of question styles and drew upon expert knowledge of the agricultural sector in England. Additional expert knowledge was gained from ‘knowledge transfer’ events with farmers such as the UK Cereals event2, the largest technology transfer event targeted at arable farmers and the cereal industry. The survey was carried out in conjunction with the Farm Business Survey (FBS) which provided additional information on farm businesses, crop yields and areas. An initial questionnaire was developed and piloted by FBS research officers (ROs) in on-farm interviews between December 2010 and February 2011, resulting in information that was incorporated into the final questionnaire. Data collection was undertaken between February 2011 and November 2011 using face-to-face on-farm interviews by the FBS ROs across England; the survey was carried out on Cereal, Mixed and General Cropping farm types (Anon, 2012d). Some of the farms surveyed in the pilot were included in the final survey results since researchers gained additional further information, required by the final survey, after the initial interview. The date each on-farm interview was conducted was recorded. Additional information on the survey design, piloting and data collection is given in Glithero et al. (in press). The survey is extensive in coverage; questions of relevance for this paper are shown in Appendix A. These relate to the price at which farmers would sell cereal straw, the amounts they would be willing to supply, the length of time they would supply straw for, and their price and straw supply quantity preferences for inclusion in an industry supply contract. In addition, information on the potential barriers to the removal of straw and incentives needed to gain this co-product from farmers was also collected. The paper relates to questions 11–18 on the farm survey; 240 completed farm returns were available for these questions and were used in the analysis presented below. The number of farms by UK Government Office Region (GOR) and farm type are given in Table 1.

### 2.2 Data analysis

Where qualitative data was collected Chi-squared tests have been performed in order test for location (GOR and EU regions), farm type and farm size effects; specifically this relates to questions 11, 12, 16 and 17. Where expected cell counts of less than five occurred, categories were combined to ensure that the assumptions of the Chi-squared test were not violated. In addition to the on-farm survey, published average farm-gate market price data for wheat straw were collected to test the hypothesis that there was no link between the responses farmers gave with respect to the minimum stated farm-gate price at which they would sell straw, and the prevailing published average farm-gate market price. Data on the area of straw that farmers would sell to a bioethanol plant was linked with the FBS data on farm cropping areas and has been aggregated to GOR level and combined with area yields from Glithero et al. (in press) to provide aggregated national supply information; the crop area aggregation method used is outlined in Glithero et al. (in press). For the purpose of aggregation, farm type and GOR combinations with fewer than five observations were combined; this was undertaken for the Mixed and General Cropping farms in the North East, North West, East of England, South East and South West. Estimates of straw availability to the market for bioenergy purposes have been calculated under two assumed scenarios, each based on farmers’ stated intentions of the amount of straw they would sell for bioenergy purposes given appropriate contract or market conditions. Under the first scenario it is assumed that for farms which currently chop and incorporate straw, the straw that would be made available for bioenergy purposes would, in the first instance, be derived from that straw which is currently incorporated, and where the stated volume to be sold exceeds that currently incorporated, it is assumed that the straw supply would then displace that currently sold or used on farm; on farms which do not incorporate straw, the straw derived for bioenergy would directly displace that currently sold or used on farm. Under the second scenario it is assumed that for farms which sell or use straw on-farm, the straw that has been stated that would be made available for bioenergy purposes would, in the first instance, be derived from that straw which is currently sold or used on-farm, and where the stated volume to be sold exceeds that currently sold or used on-farm, it is assumed that the straw supply would then displace that currently incorporated.

Analysis of regional straw travel distances has also been undertaken. For the three main cereal straw producing GORs of England, a potential location for lignocellulosic bioethanol plants was proposed towards the centre of the cereal growing areas within each GOR, based on population crop maps (Defra, 2011). The Northern, Eastern, Southern and Western maximum major road distances from this proposed plant location were then calculated using a standard road distance on-line calculator (Google, 2013) in order to establish the logistical potential for regional bioethanol plants based on the volumes of straw available in these main straw-producing GORs.

## 3 Results

### 3.1 Length of supply and length of contract

Farmers were asked to state the maximum length of time (in years) they would consecutively supply straw, to a bioenergy plant, and also the maximum contract length that would be acceptable to them. The most popular contract lengths were 3 years (23%), 1 year (22%) and ‘none’ (20%). The most popular number of consecutive years of supply was none (24%), followed by 1 year (18%) and 3 years (17%). The majority (71%) of farmers cited the same response for both the consecutive number of years of supply and maximum contract length, as indicated by the diagonal percentage responses in Table 2. Note that whilst contract lengths of 3 years or less were most popular, 5 year contracts with 5 years of consecutive supply was also cited by 10%, and 14% would supply for 15 consecutive years, albeit that the majority of these respondents would not wish to agree to a contract length of 15 years duration.

### 3.2 Contract volumes and farm-gate price preferences

**Fig. 1:**
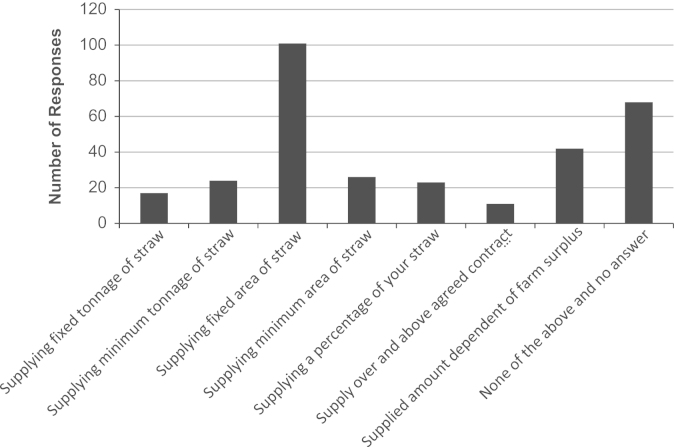
Quantity supply contract option preferences.

**Fig. 2:**
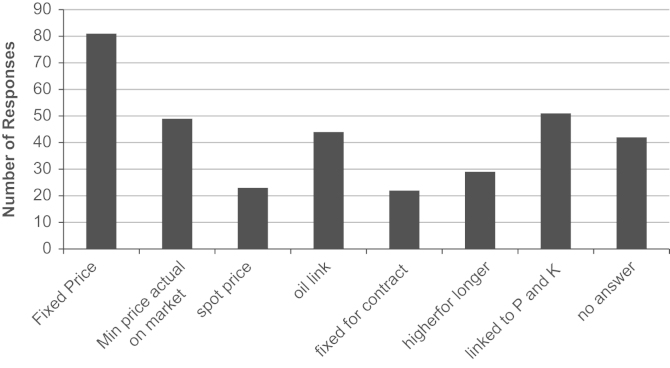
Farm-gate price supply contract option preferences.

Farmers’ contract condition preferences in terms of stated farm-gate price and quantity options for straw sold via a contract with a bioethanol plant were obtained in Questions 17 and 16. From a variety of possible contract options, farmers registered an interest against as many options as they wished. The most popular responses (Fig. 1) were recorded as supplying a fixed area of straw (42% of farmers), for a fixed farm-gate price (34% of farmers). Using a Chi-squared test, these data were analysed with respect to farm type (Cereal, General Cropping and Mixed), farm size (Small, Medium and Large) and EU region (North England, West England and East England) to test the hypothesis of no influence of farm type (price, *p*=0.31; quantity, *p*=0.41), farm size (price, *p*=0.37; quantity, *p*=0.022) and EU region (price, *p*=0.41; quantity, *p*=0.002) on farm-gate price and quantity responses. Hence, whilst no significant impact of farm type, size and region was observed for contract farm-gate price options, there is a significant impact of farm size and EU region on the preference for quantity options within contracts. Approximately one-third of cereal (35%) and general cropping (37%) farmers would supply a fixed area of straw; however, approximately one-fifth (22%) of mixed farm farmers would find this option acceptable. Supplying a fixed area of straw was the most popular choice; East England farmers were almost twice as likely as North England farmers to choose this option (40% and 21% respectively). In addition to preferring fixed farm-gate price contracts, farm-gate straw prices linked to P and K inputs, receiving a guaranteed minimum farm-gate price with actual price based on market forces and a farm-gate price based on oil prices were also popular responses (Fig. 2).

### 3.3 The farm-gate price of straw

**Fig. 3:**
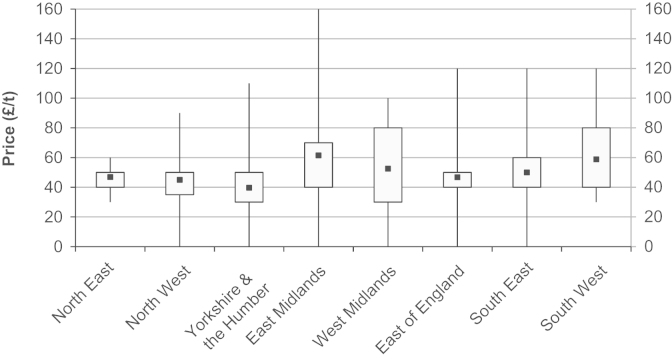
Minimum farm-gate price for wheat straw by Government Office Region. The boxes represent the 25% and 75% quartiles with the whiskers showing the full extent of the data.

**Fig. 4:**
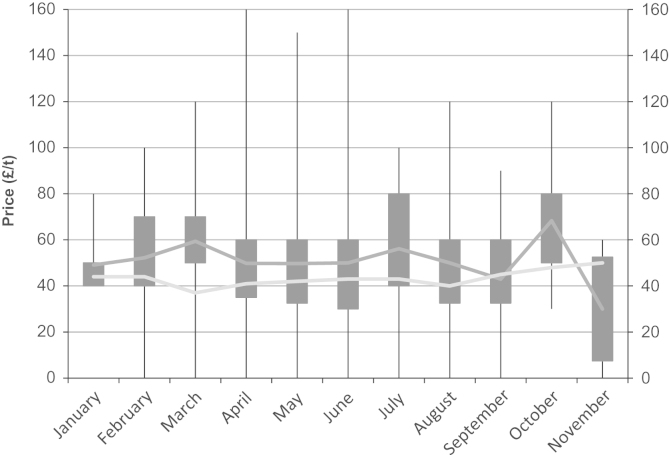
Minimum farm-gate price for wheat straw and prevailing farm-gate market price over survey period. The boxes represent the 25% and 75% quartiles with the whiskers showing the full extent of the data. The dark grey line represents the mean of the data and the light grey line represents the published average farm-gate market price data from Defra.

The minimum farm-gate price per tonne that farmers stated that they would be willing to sell wheat straw for was collected to indicate the farm-gate prices that would be potentially required in contracts in order to attract supplies of wheat straw to a bioenergy plant. The most frequent farm-gate price cited was £50 t^−1^ selected by 24% of all respondents irrespective of whether they would actually sell straw (Question 13). Additionally, of the 157 respondents that would be willing to sell their wheat straw to a bioenergy plant, the most popular farm-gate price cited was also £50 t^−1^ selected by 27% of the ‘willing to sell’ respondents. The variability in the farm-gate price response data can be observed when linked to GORs (Fig. 3); there appears to be no East–West differentiation given that the highest mean prices were observed in the East Midlands and the South West. Examining the farm-gate price of straw against the month in which the interview took place (Fig. 4) highlights the wide spread of price responses. The mean stated farm-gate price per month from the survey results was compared to the Defra quoted published average farm-gate market price for straw for that month, for England and Wales, and no correlation was found between minimum stated price response from the survey and prevailing published average market price (Spearman's rank correlation coefficient=−0.314, *p*=0.082).

### 3.4 Reasons for not baling straw and incentives to encourage baling

**Fig. 5:**
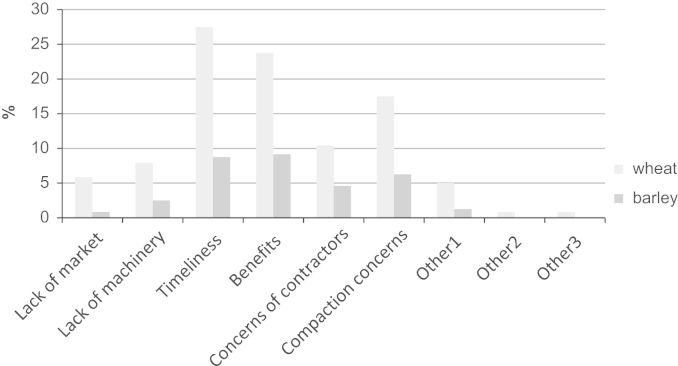
Reasons for not baling straw.

**Fig. 6:**
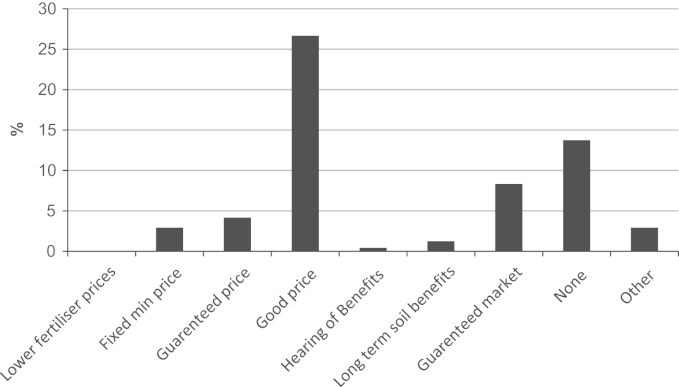
Incentives to encourage straw baling.

Where farmers did not bale, or sell in swath, some or all of their straw, reasons for not baling were obtained. In the case of wheat (Fig. 5), 28% of all farmers stated that timeliness of operations (i.e. delays in establishment of the next crop) was the reason for not baling (including selling in swath) with 24% stating perceived benefits of incorporation (e.g. soil structure/nutrients). These two reasons were also the most popular responses for barley. The question allowed farmers to provide additional reasons for not baling which generally fell into the following groupings: weather (e.g. difficulty in achieving dry straw to bale), fertiliser (e.g. specifying particular nutrient benefits), arson (e.g. concern about security of harvested straw), neighbour or market (e.g. lack of local interest in straw), price related (e.g. returns insufficient), time/labour/complexity of farm operations (e.g. need to invest in new machinery and would require labour at busy period of year). In addition farmers were also asked what single factor would most encourage them to bale their straw (Fig. 6); a ‘good price’ for straw sold at the farm gate was given by 26% of farmers.

### 3.5 Potential cereal straw supply and regional lignocellulosic bioethanol analyses

Given acceptable market or contract conditions and based on preferences expressed in the survey, the supply of straw that farmers would be willing to sell for bioenergy purposes is presented in Table 3, based on the mean straw yields cited in Glithero et al. (in press). Of the total 2.52 Mt of cereal straw available, 1.99 Mt is from wheat with the remaining 529,000 t from barley; however Glithero et al. (in press) note the potential variation that exists in per hectare straw yields.^3^ The East of England contains the largest area of cereals, of both wheat and barley; however, it records the lowest (barley straw) and second lowest (wheat straw) percentage of production that farmers would be willing to supply for bioenergy purposes, at 21% and 43% respectively. Despite this, the survey results indicate that the East of England would still supply 346 kt of wheat straw and 47 kt of barley straw for bioenergy. The East Midlands contains the greatest potential straw for use/sale for bioenergy purposes (wheat 686 kt; barley 146 kt) with Yorkshire and Humber producing a potential supply of 271 kt and 150 kt of wheat and barley straw respectively. Overall, we calculate that approximately 48% of the cereal straw produced in England on arable farm types would be available for sale for bioenergy production. Conversely, 35% of farmers surveyed said they would not supply wheat straw for bioenergy; this rises to 64% for barley straw. Overall, 31% of farmers would supply neither type of straw for bioenergy.

While Table 3 provides a national assessment of potential cereal straw supply, it is recognised that the low-value nature of the feedstock will lead to regional restrictions on the demand for the feedstock as bioethanol plants seek to achieve sufficient feedstock supply while reducing the transport distances involved. Dunnett et al. (2008) provide a spatial assessment of lignocellulosic bioethanol processing potential in a European context and note maximum feedstock delivery distances of 140 km under current technology, reducing to an average of 66 km under a future technology modelling scenario. Within a US context Brechbill et al. (2011) examined biomass collection for cellulosic facilities within a 10–100 km distance range. Location specific sites, and associated potential feedstock supply are therefore of direct interest. Table 4 presents a summary of the total potential cereal straw supply for the three largest straw supplying GORs from Table 3, together with potential locations for a lignocellulosic bioethanol plant within each GOR and the maximum major road distances from each plant location to the outer boundaries of the GOR. Given the potential supply in these regions, with assumed ethanol yields of 290–333 l ethanol/t of straw (Brechbill et al., 2011; Glithero et al., in press respectively), the Yorkshire and Humberside, East Midlands and East of England GORs could supply three independent lignocellulosic bioethanol plants respectively producing 123–140 million litres (Ml), 241–277 Ml and 114–131 Ml annually.

#### 3.5.1 Straw supply primarily derived from straw incorporated and secondly from straw sold or used on-farm

**Fig. 7:**
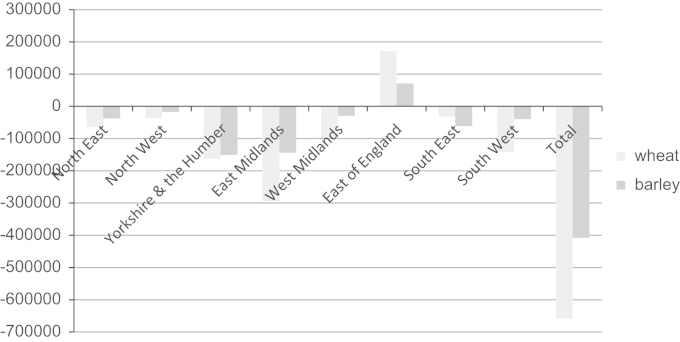
Incorporated straw (tonnes) net of potential straw for bioenergy. Incorporated straw values from Glithero et al. (in press); potential bioenergy supply as in Table 3.

Fig. 7 combines the results from Table 3 with those in Glithero et al. (in press). It can be seen that only the East of England would be able to supply all the cereal straw, both wheat and barley, for lignocellulosic bioethanol production from that which is currently incorporated into the soil. In all other GORs the amount that farmers indicated that they would sell for bioenergy exceeds that which is currently chopped and incorporated, albeit that the low overall volumes of potential supply in some GORs would not be economical to transport to a commercially viable lignocellulosic bioethanol plant. However, overall, the deficit for England, of potential supply over that which is incorporated, is 1 Mt of cereal straw, wheat and barley combined (assuming 1.5 Mt currently chopped and incorporated calculated by Glithero et al., in press).

Table 5 presents estimates of straw availability from farmers who currently incorporate some or all their cereal straw and that have indicated that they would be willing to sell straw for bioenergy purposes. Total straw chopped and incorporated was estimated to be approximately 1.5 Mt, with approximately 840,000 t available for sale across all the GORs of England. Overall, 57% of the total straw chopped and incorporated is estimated to be available for sale for bioenergy purposes, on the assumption that straw sales for bioenergy from farms that chop and incorporate straw would be met first from straw that is currently incorporated.

#### 3.5.2 Straw supply primarily derived from straw sold or used on-farm and secondly from incorporated straw

Based on farmer responses from the survey, Table 6 shows the amount of straw that would be sold for bioenergy assuming that this market is met first from straw currently used on-farm or sold, and second from straw currently incorporated. Nationally, of the current straw sold or used on farm, 1.965 Mt of cereal straw would be made available for bioenergy purposes under acceptable contract conditions, diverting 52% of straw away from current uses. In the East of England farmers’ willingness to supply to a bioethanol plant would lead to 43% (20%) of the wheat (barley) straw from current markets being diverted to bioethanol production. In the East Midlands the percentage of straw diverted from current markets would be 68% for both wheat and barley. In Yorkshire and the Humber, these respective estimates are 51% and 55%. Hence, in the three GORs with the largest potential to supply cereal straw for bioenergy purposes, the cited quantities that farmers would be willing to supply account for 20–68% of the specific cereal straw market or on-farm use for that GOR.

**Fig. 8:**
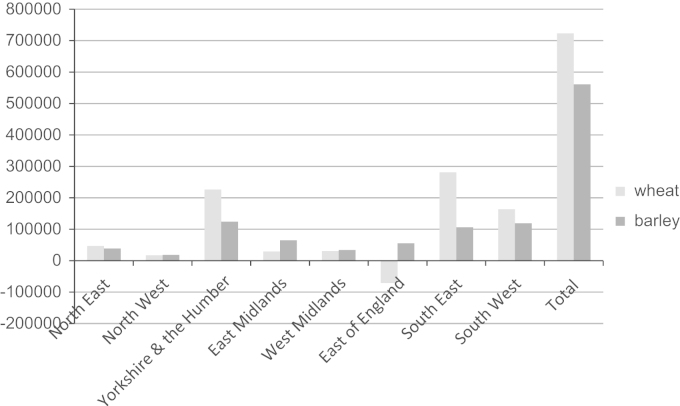
Straw sold or used on-farm (tonnes) net of potential straw for bioenergy. Total straw used and total straw that would be sold for bioenergy from Table 6.

Fig. 8 shows straw currently sold or used on farm less that which would be supplied for bioenergy purposes. It can be seen that for wheat straw in the East of England the amount of straw currently sold or used on farm is insufficient to fulfil the stated straw volumes that farmers would be willing to supply to a bioenergy plant. In total, under the assumption that straw supply for bioenergy purposes would be derived first from straw currently sold or used on-farm, this would result in 723 kt (561 kt) of wheat (barley) straw being retained for current markets or on-farm use, in comparison to 2.71 Mt (1.09 Mt) currently sold or used on-farm.

## 4 Discussion

The results indicate that farmers on arable farm types in England would potentially sell 2.5 Mt of cereal straw for bioenergy purposes. However, examining regional prospects for lignocellulosic bioethanol plants and associated transport distances, it is apparent that there is sufficient cereal straw potential in only three regions of England: the GORs of the Yorkshire and Humber, East Midlands and East of England could potentially provide 1.65 Mt of cereal straw to produce 478–549 Ml of bioethanol. However, on the basis of the estimated levels of ethanol production in the Yorkshire and Humber (123–140 Ml) and East of England (114–131 Ml), production in independent smaller plants may be commercially unviable; an alternative scenario would be to establish a plant near the border of the East Midlands and the East of England GORs, to gain sufficient industrial scale in a single bioethanol (355–408 Ml) plant for these two GORs only. This would lead to longer average transport distances which may be accommodated by a larger bioethanol plant benefiting from greater economies of scale in overall production, albeit that crucial feedstock distance-to-plant scale considerations will be central to commercial success (Dunnett et al., 2008). However, there is also potential for increased yields of cereal straw to be generated from the East of England (Glithero et al., in press) via changes in crop varietal choice, crop management techniques and crop harvesting height. Moreover, the estimates for straw production in the East of England are based on the 2010 harvest, which has been noted to be associated with lower than anticipated straw yields due to periods of dry weather in the early crop growing season (Horne, 2010).

A third of the farmers interviewed said that they would not supply wheat straw and just under two-thirds would not supply barley straw for bioethanol purposes. Many farmers that currently chop and incorporate their straw are not willing to bale straw for bioenergy purposes, as indicated by the 43% of chopped and incorporated straw that farmers would not sell for bioenergy. On the basis of the estimates produced, for the three GORs of Yorkshire and Humber, East Midlands and East of England combined, 1.65 Mt of cereal straw would potentially be made available for bioenergy purposes. Assuming this potential straw supply is met first from straw that is currently incorporated, 652 kt could be derived from this source. This accounts for 40% of the total straw estimated to be available for bioenergy purposes. Alternatively, assuming that the stated bioenergy straw supply would be met first from straw that is currently sold or used on-farm, 1.17 Mt of cereal straw would be diverted from these current uses, equating to 56% of straw harvested within these GORs and sold or used on-farm. Consequently, bioenergy production from cereal straw is likely to have a significant impact on current straw markets, and hence the market price for straw, in turn affecting the financial viability of feedstock supply for bioenergy purposes.

Barriers to baling straw vary slightly between wheat and barley crops but mainly relate to the timeliness of operations in establishing the next crop (Darby and Yeoman, 1994) and the perceived benefits of straw incorporation to soil properties (Cherubinia and Ulgiatib, 2010; Lal, 2008). The timeliness of operations is dependent on the crop rotations chosen by the farm business as well as weather considerations (Yamoah et al., 1998). Advances in crop developments may allow for later sowing dates, partially negating concerns over timeliness; however, research suggests that earlier sowing dates are associated with increased straw yields in winter wheat (Donaldson et al., 2001). The benefits of straw incorporation could potentially be addressed if the process residue from bioethanol production had nutrient and soil structure benefits when applied to land. Digestate from anaerobic digestion, usually producing biogas, can have good nutrient content and is a good replacement for inorganic fertilisers (Tambone et al., 2010). If mechanisms can be put in place to return biological digestate from bioenergy processes, replacing nutrients lost in straw removal, these could form part of contractual agreements between farm businesses and bioenergy/bioethanol producers.

In relation to the contractual implications of bioethanol production straw-feedstock supply, short-term contracts were typically favoured and the majority would consider supplying straw for only the same length of time as the contract. For large scale investment in bioenergy from cereal straw, security of feedstock supply will be needed; farmer responses indicate that security of a fixed farm-gate price and supplying a fixed area were preferred contract options. No relationship was found between the published average farm-gate market price for wheat straw at the time of the interview and the prices at which farmers said they would sell their wheat straw. However, the preferred stated farm-gate straw price of £50 t^−1^ is high in comparison to that provided by the EPR Ely power station which paid £35 t^−1^ for half tonne Hesston bales supplied to the plant gate and £2 t^−1^ when ‘sold in swath’ (Anon, 2004). However, farm-gate straw prices for big square bales have increased by 178% (£18–£50 t^−1^) and 154% (£24–£61 t^−1^) for wheat and barley respectively between January 2004 and November 2011 (authors calculation based on Defra, 2012), indicating the change in market conditions over this time period. In contrast, contracts and prices for dedicated energy crops are constantly being updated to reflect the market with the introduction of 5–10 year index-linked contracts being introduced in the UK (Wragg, 2011; Spackman, 2012). Index linking straw prices may also offer potential incentives to farmers; the second most popular contract supply option cited was for the farm-gate price of straw to be linked to P and K prices (the nutrients supplied by straw when chopped and incorporated), while farm-gate straw prices linked to the oil price was the fourth most popular contract option noted by farmers in the survey. Such approaches would directly address issues of specific (soil nutrient) and general (fuel, nitrogen fertiliser) cereal production costs. Moreover, introducing longer term index-linked contracts as noted for bioenergy crops would arguably increase willingness of farmers to supply cereal straw.

While government support policies are in place for dedicated energy crops, no policies relating to straw use for bioenergy purposes exist. The results presented indicate that potentially 44% of straw currently used for other purposes could be sold for bioenergy, while 57% of the straw that is currently chopped and incorporated would be made available for bioenergy purposes, the latter representing only one-third of the estimated straw that farmers were willing to sell for bioenergy purposes. Hence, large scale bioenergy production using cereal straw as a feedstock will have a large impact on the market price for cereal straw, potentially detracting from the commercial viability of bioenergy from cereal straw feedstock. Given that policies exist to incentivise farm-level production of dedicated energy crops, policy makers could consider incentives for cereal straw supply for bioenergy purposes; the challenge will be to provide these incentives for feedstock supply in a manner that minimises disruption to current straw markets, and additionally takes a holistic view of arable soil structure and nutrient management aspects without impacting upon food supply. Alternatively, support for dedicated energy crops could be reduced, to create a ‘level playing field’. While policy incentives alone may not encourage straw sales, as observed with incentives for establishing dedicated energy crops (Sherrington and Moran, 2010), it is argued that incentivising farmers to supply a co-product from widely grown food crops would be met with greater uptake by the agricultural sector than for dedicated energy crops. Such policy intervention may require stabilisation of feedstock markets in conjunction with more rational levels of support for feedstock derived from dedicated energy crops and that provided from co-products. However, it is worth noting that the UK Code of Good Agricultural Practice (Anon, 2009a) recommends straw incorporation; to achieve both fuel and food security, policy messages must therefore be increasingly integrated to address the potential dichotomy of encouraging bioenergy production without overly compromising food production and thus exacerbating concerns relating to food security (Subhadra and Grinson, 2011).

## 5 Conclusion

While first generation technologies have thus far been at the forefront of bioenergy production, concerns relating to land use conflict together with investment in technological developments in second generation biofuels are changing the outlook for bioenergy products. Securing feedstock supply for bioenergy represents a necessary condition if second generation technologies are to play a part in meeting the EU target for renewable fuel use. Second generation biofuel feedstock potentially includes dedicated energy crops and agricultural crop residues, in particular, in the UK context, from cereal straw. From an extensive, on-farm survey of 240 arable farmers, we have identified that while barriers to the use of straw for bioenergy exist, two-thirds and one-third of farmers would respectively be willing to supply wheat and barley straw for bioenergy purposes. In addition, a range of contract preferences have been identified that are of direct interest to both the fuel industry and policy makers alike. The farm-gate market price for baled straw at which farmers would be willing to supply cereal straw currently exceeds that obtained by farmers supplying baled straw for current large scale industrial energy use. Thus, policy interventions in the market for straw as a bioenergy feedstock may be required in order to further incentivise farmers to engage in this potentially new market. However, farmers’ attitudes towards straw removal or incorporation are potentially well-founded with respect to the perceived benefits of straw incorporation in maintaining soil quality, nutrient retention and providing timeliness of crop establishment to ensure both immediate and long term crop productivity on their land. Hence policy incentives towards bioenergy production must be increasingly integrated with the sustainability of food supply if policy makers are to achieve the combined goals of food and fuel security.

## Figures and Tables

**Table A1:** If ALL straw (for all three crops mentioned) is currently baled on farm by you, by a third party (e.g. sold in swath) or by a contractor then proceed to Question 13 if not got to Question 11.

**11**	**If you do not currently bale some/all of your straw, tick all applicable reasons for not doing so for the relevant crop(s)?**		
	Lack of a market		
	Lack of machinery		
	Timeliness of operations (i.e. Delays in establishment of the next crop)		
	Perceived benefits of incorporation (e.g. soil structure/nutrients)		
	Concerns about using contractors or selling in swath		
	Concerns about soil compaction		
	Other (please state)		
	Other (please state)		
	Other (please state)		

**12**	**What single factor would most encourage you to bale straw?**		
			If other please state
			1. ***Lower fertiliser prices*** 2. ***Fixed minimum price for the straw*** 3. ***Guaranteed price*** 4. ***Good price*** 5. ***Hearing the benefits from other farmers*** 6. ***Long term soil benefits*** 7. ***Guaranteed market*** 8. ***None*** 9. ***Other***

**13**	**What is the lowest price you would be willing to sell your baled wheat straw for? (£/t)**		
	**This is the selling price for big bales sold off the field/at farm gate assuming typical harvest and current costs**		

**14**	**If there was a market for using straw in a bioenergy power plant what percentage of your straw would you be willing to sell if the price was acceptable to you?**		

**15**	**What is the number of consecutive years you would supply cereal straw for (for bioethanol production)?**		

**16**	**Which type of contract volumes would you be willing to supply straw to a bioethanol power plant (tick all appropriate)**		
	Supplying fixed tonnage of straw		
	Supplying minimum tonnage of straw		
	Supplying fixed area of straw		
	Supplying minimum area of straw		
	Supplying a percentage of your straw		
	Supply over and above agreed contract amount		
	Supplied amount dependent of farm surplus		
	None of the above		

**17**	**Which types of straw prices would you prefer in a contract to supply a bioethanol power plant (tick all appropriate)**		
	Fixed price		
	Minimum price with actual price based on market forces		
	Spot market price		
	Price linked to the price of oil		
	Fixed price for contracted tonnage with spot market price for supply beyond this		
	Higher price for longer term contracted supply		
	Price linked to prices of P and K fertilisers		

**18**	**What is the maximum length of contract you would find acceptable (if contract agreements were acceptable to you)? (years)**		

**Table 1:** Number of survey respondents by farm type and government office region.

GOR	Cereals	General cropping	Mixed
North East	8	1	7
North West	7	5	4
Yorkshire and Humber	13	5	11
East Midlands	31	9	7
West Midlands	5	8	7
East of England	29	21	4
South East	20	3	9
South West	11	4	11

**Table 2:** Percentage response for consecutive number of years supplying and maximum contract length.

Consecutive years willing to supply	Maximum contract length											
	0	1	2	3	4	5	6	7	8	9	10	15
0	19.0	2.1	0.8	1.7								
1		15.0	2.1	0.4								
2			5.8	0.8	0.4	0.4						
3			1.3	15.0	0.4	0.4						
4			0.8		0.8	0.4						
5	0.4	0.4	1.3	2.1	0.4	10.0					0.4	
6												
7								0.4			0.4	
8												
9												
10		0.4	0.4		0.4						0.8	
15		3.3	1.7	2.5		2.1					0.8	3.3

**Table 3:** Area, yields and potential supply of wheat and barley straw; GOR area and associated straw yields taken from Glithero et al. (in press).

Crop	GOR	Area in GOR	Straw yield	Potential total straw (t)^a^	Potential supply to bioenergy (t)^b^	Percentage supply of total^c^
Wheat	North East	62,021	2.52	156,114	87,054	55.76
	North West	24,066	2.21	53,093	35,760	67.35
	Yorkshire & the Humber	220,285	2.76	606,894	271,737	44.77
	East Midlands	340,059	3.26	1,108,195	685,874	61.89
	West Midlands	147,223	1.88	277,353	172,706	62.27
	East of England	482,895	1.66	800,943	345,843	43.18
	South East	222,206	3.34	741,744	246,356	33.21
	South West	136,923	2.23	305,467	141,804	46.42
	Total	1,635,678	2.48	4,049,803	1,987,135	49.07

Barley	North East	32,132	2.38	76,475	37,677	49.27
	North West	18,328	2.00	36,647	17,607	48.04
	Yorkshire & the Humber	90,258	3.04	274,486	150,525	54.84
	East Midlands	59,692	3.58	213,753	146,232	68.41
	West Midlands	35,096	1.81	63,449	29,364	46.28
	East of England	118,475	1.95	230,685	47,331	20.52
	South East	57,252	2.92	167,090	60,948	36.48
	South West	70,611	2.25	158,641	39,538	24.92
	Total	481,845	2.53	1,221,228	529,221	43.34
**Cereals total**		2,117,523		5,271,031	2,516,356	47.74

a Area in GOR multiplied by straw yield.

b Per farm crop areas multiplied by the percentage of straw would be willing to sell for bioenergy multiplied by the regional straw yield, aggregated to GOR levels (method cited in Glithero et al., in press).

c Potential supply to bioenergy as a percentage of potential total straw.

**Table 4:** Potential supply of cereal straw, bioethanol plant locations and maximum major road distances (km) from plant to major cereal growing areas for three GORs with substantial cereal straw supply.

GOR	Potential cereal supply to bioenergy (t)^a^	Potential plant location	Maximum major road distance from potential plant location to major cereal growing areas (approximate km)^b^			
			North	East	South	West
Yorkshire & the Humber	422,262	York (North of City)	76	66	58	34
East Midlands	832,106	Lincoln	37	68	66	41
East of England	393,174	Bury St Edmunds	104	84	116	82

a Derived from Table 3.

b Calculated using Google Maps (Google, 2013) informed by wheat and barley crop maps from Agriculture in the UK (Defra, 2011).

**Table 5:** Estimated straw incorporated and proportion of incorporated straw that would be available for bioenergy purposes by crop type and Government Office Region; GOR area and associated straw yields taken from Glithero et al. (in press).

Crop	GOR	Area in GOR	Straw yield	Total straw chopped^a^	Total straw chopped but would be sold^b^	Percentage of total chopped straw that would be sold^c^
Wheat	North East	62,021	2.52	22,425	12,527	55.86
	North West	24,066	2.21	0	0	
	Yorkshire & the Humber	220,285	2.76	108,999	45,162	41.43
	East Midlands	340,059	3.26	393,044	297,644	75.73
	West Midlands	147,223	1.88	74,139	74,139	100.00
	East of England	482,895	1.66	526,208	279,725	53.16
	South East	222,206	3.34	214,589	98,794	46.04
	South West	136,923	2.23	0	0	
	Total	1,635,678	2.48	1,339,403	807,991	60.32

Barley	North East	32,132	2.38	0	0	
	North West	18,328	2.00	594	450	75.76
	Yorkshire & the Humber	90,258	3.04	0	0	
	East Midlands	59,692	3.58	2,530	2,530	100.00
	West Midlands	35,096	1.81	0	0	
	East of England	118,475	1.95	127,890	27,160	21.24
	South East	57,252	2.92	0	0	
	South West	70,611	2.25	0	0	
	Total	481,845	2.53	131,014	30,139	23.00
Cereals total		2,117,523		1,470,417	838,130	57.00

a Per farm crop areas multiplied by the percentage of area that would be chopped and incorporated multiplied by the regional straw yield, aggregated to GOR levels (method cited in Glithero et al., in press).

b Per farm minimum value of either the area of straw chopped or the area of straw that farmers would be willing to be sell for bioenergy, multiplied by the regional straw yield, aggregated to GOR levels (method cited in Glithero et al., in press).

c Total straw chopped but would be sold as a percentage of total straw chopped.

**Table 6:** Estimated straw usage and the proportion of used straw that would be available for bioenergy purposes by crop type and Government Office Region; GOR area and associated straw yields taken from Glithero et al. (in press).

Crop	GOR	Area in GOR	Straw yield	Total straw used a	Total straw used but would be sold b	Percentage of total used straw that would be sold c
Wheat	North East	62,021	2.52	133,689	80,758	60.41
	North West	24,066	2.21	53,093	35,760	67.35
	Yorkshire & the Humber	220,285	2.76	497,895	251,837	50.58
	East Midlands	340,059	3.26	715,151	482,894	67.52
	West Midlands	147,223	1.88	203,215	139,666	68.73
	East of England	482,895	1.66	274,735	118,141	43.00
	South East	222,206	3.34	527,155	214,867	40.76
	South West	136,923	2.23	305,467	141,804	46.42
	Total	1,635,678	2.48	2,710,400	1,465,727	54.08

Barley	North East	32,132	2.38	76,475	37,677	49.27
	North West	18,328	2.00	36,053	17,463	48.44
	Yorkshire & the Humber	90,258	3.04	274,486	150,525	54.84
	East Midlands	59,692	3.58	211,224	143,702	68.03
	West Midlands	35,096	1.81	63,449	29,364	46.28
	East of England	118,475	1.95	102,795	20,172	19.62
	South East	57,252	2.92	167,090	60,948	36.48
	South West	70,611	2.25	158,641	39,538	24.92
	Total	481,845	2.53	1,090,214	499,388	45.81
Cereals total		2,117,523		3,800,613	1,965,115	51.71

a Area in GOR multiplied by the straw yield minus the total straw chopped from Table 5.

b Per farm minimum value of either the area of straw used or the area of straw that farmers would be willing to sell for bioenergy, multiplied by the regional straw yield, aggregated to GOR levels (method cited in Glithero et al., in press).

c Total straw used that farmers would be willing to sell for bioenergy as a percentage of the total straw used.

## Appendix A

See Table A1.

## Footnotes

1 Specifically, sold-in-swath refers to straw sales where the straw purchaser undertakes to bale and removes the straw from the field directly following the harvest of the crop from which the straw is left in rows (swaths) in the field. This is in contrast to the farmer baling the straw and selling the produce as a baled commodity.

2 The UK Cereals event describes itself as ‘the leading technical event for the UK arable industry’ with approximately 26,900 visitors annually.

3 Glithero et al. (in press) show 95% confidence intervals for the median straw yields for wheat and barley; the upper bound for wheat (barley) being 21% (18%) above the median value and the lower bound being 8.7% (8.4%) below.
